# Curcumin and Methotrexate: A Promising Combination for Osteosarcoma Treatment via Hedgehog Pathway Inhibition

**DOI:** 10.3390/ijms252011300

**Published:** 2024-10-21

**Authors:** Giulia Giliberti, Maria Maddalena Marrapodi, Giuseppe Di Feo, Elvira Pota, Martina Di Martino, Daniela Di Pinto, Francesca Rossi, Alessandra Di Paola

**Affiliations:** 1 Department of Experimental Medicine, University of Campania “Luigi Vanvitelli”, 80138 Napoli, Italy; giulia.giliberti@unicampania.it; 2 Department of Woman, Child and General and Specialist Surgery, University of Campania “Luigi Vanvitelli”, 80138 Napoli, Italy; mariamaddalena.marrapodi@unicampania.it (M.M.M.); giuseppe.difeo.bio@gmail.com (G.D.F.); elvira.pota@gmail.com (E.P.); martina.dimartino@policliniconapoli.it (M.D.M.); daniela.dipinto@policliniconapoli.it (D.D.P.); alessandra.dipaola@unicampania.it (A.D.P.); 3 Department of Life Sciences, Health and Health Professions, Link Campus University, 00165 Rome, Italy

**Keywords:** osteosarcoma, curcumin, methotrexate, hedgehog signaling pathway, therapeutic targets

## Abstract

Osteosarcoma (OS) is the most severe bone tumor in children. A chemotherapy regimen includes a combination of high-dose Methotrexate (MTX), doxorubicin, and cisplatin. These drugs cause acute and chronic side effects, such as infections, thrombocytopenia, neutropenia, DNA damage, and inflammation. Therefore, to identify new therapeutic strategies, effective and with a safety profile, is necessary. The Hedgehog (Hh) signaling pathway involved in tumorigenesis is active in OS. Hh components Patched receptor 1 (PTCH1), Smoothened (SMO), and glioma-associated oncogene homolog transcription factors (GLI1 and GLI2) are overexpressed in OS cell lines and patient samples. Curcumin (CUR)—with antioxidant and anti-cancer properties—downregulates Hh components in cancer, inhibiting progression. This study investigates CUR effects on the MG-63 OS cell line, alone and combined with MTX, to propose a novel therapeutic approach. Our study suggests CUR as a novel therapeutic agent in OS, particularly when combined with MTX. Targeting the Hh signaling pathway, CUR and MTX showed significant pro-apoptotic effects, increasing the BAX/Bcl-2 ratio and total apoptotic cell percentage. They reduced the expression of Hh pathway components (PTCH1, SMO, GLI1, and GLI2), inhibiting OS cell proliferation, survival, and invasion. CUR and MTX combined determined a β-Catenin decrease and a trend toward reducing NF-kB and matrix metalloproteinases (MMP-2 and MMP-9). Our findings suggest CUR as a support to OS treatment, improving outcomes and reducing the adverse effects of current therapies.

## 1. Introduction

Osteosarcoma (OS) is the most common and severe primary malignant bone tumor, characterized by a bimodal incidence pattern: its first peak appears in children—as a primary bone cancer—and the second in adults—as a secondary cancer linked to radiation therapy or other conditions [1,2,3,4].

It predominantly occurs in the long lower bones, carrying a significant risk of spreading to other bones and metastasizing preferentially in the lungs [1,2]. Although OS accounts for only 5% of tumors in pediatric patients, its severity and early metastatic potential contribute to a high mortality rate related to cancer [1,2,3]. The five-year survival rate for patients with localized OS is approximately 60–70%, whereas it drops to less than 20% for those with metastatic OS. Chemotherapy—while essential for treatment—has a severe impact on bone metabolism and physiology, resulting in osteoporosis (OP) and a decrease in bone mineral density in OS patients, making long-term survivors more susceptible to bone fractures [5].

The current standard treatment approach for OS involves neoadjuvant chemotherapy (before surgery), surgical resection (either limb amputation or more commonly limb-sparing surgery), and adjuvant chemotherapy (after surgery) [6,7]. The most common chemotherapy regimen for children includes a combination of high-dose Methotrexate (MTX), doxorubicin, and cisplatin (MAP) [8,9]. OS is marked by significant heterogeneity, resistance to chemotherapy, and intricate interactions with the surrounding bone microenvironment, making existing chemotherapeutic drugs not entirely effective [6,9,10]. Additionally, the required high doses of anti-cancer drugs often result in numerous acute and chronic side effects. Acute adverse reactions mainly include several infections, thrombocytopenia, and neutropenia [11]. However, chemotherapy is responsible not only for acute short-term side effects, but also for chronic, long-term adverse reactions, causing the onset of a low-grade chronic inflammatory state, known as inflamm-aging, which leads to the onset of age-related diseases such as osteoporosis, obesity, infertility, and cardiovascular diseases [12]. Given the limitations of current therapies, there is an urgent need to identify new therapeutic strategies that are both effective and have a better safety profile. One of the promising areas of research involves targeting specific molecular pathways that are dysregulated in OS. Among these, the Hedgehog (Hh) signaling pathway has emerged as a critical regulator of cancer cell proliferation, survival, and metastasis [13].

This pathway is a key regulatory cascade involved in embryogenesis, organogenesis, and the maintenance of tissue homeostasis [14]. It comprises ligands such as the Sonic Hedgehog (SHh), Indian Hedgehog (IHh), and Desert Hedgehog (DHh), which bind to the Patched receptors (PTCH1 and PTCH2) [15]. In the absence of ligands, PTCH inhibits Smoothened (SMO) [16]. Ligand binding leads to the internalization and degradation of PTCH, allowing SMO activation [16,17]. This triggers a signaling cascade that results in the translocation of glioma-associated oncogene homolog (GLI) transcription factors (GLI1, GLI2, GLI3) into the nucleus, where they regulate the expression of target genes involved in cell proliferation, differentiation, and survival [15].

Aberrations in Hh signaling are implicated in various cancers, including leukemia, basal cell carcinoma, and medulloblastoma [13,18]. Dysregulation of this pathway contributes to tumorigenesis by promoting cell proliferation, enhancing resistance to apoptosis, and facilitating DNA damage repair. GLI1, a key transcription factor in the Hh pathway, is particularly important for mediating these oncogenic processes [13,19,20].

In OS, the Hh pathway plays a significant role in disease progression and metastasis [21]. Studies have shown that components of the Hh pathway, such as SHh, PTCH1, SMO, and GLI1/GLI2, are overexpressed in OS cell lines—143B, SaOS2, KHOS, and U2OS—and patient samples [21,22,23,24]. This overexpression correlates with poor clinical outcomes, as the activation of the Hh pathway promotes the cell cycle progression, proliferation, and survival of OS cells [25]. GLI1 overexpression, in particular, has been linked to increased metastatic potential and chemoresistance, making the Hh pathway a promising target for therapeutic intervention in OS [13,19,20]. In particular, it has been documented that GLI1 expression is elevated in OS patients and in cells resistant to cisplatin [26]. Conversely, inhibiting GLI1 expression significantly restores the sensitivity of OS cells to cisplatin, reduces the proliferation, migration, and cloning abilities of both cisplatin-sensitive and cisplatin-resistant cells, and increases the apoptosis rate in vitro [13]. However, Lo et al. demonstrated that patients with elevated GLI1 levels respond positively to chemotherapy [23,24]. Moreover, it has also been reported that GLI2 overexpression is associated with poor clinical prognosis in OS, predisposing patients to lung metastasis and poor clinical outcomes, thus contributing to OS progression [27,28]. Interestingly, the inhibition of both GLI1 and GLI2 suppresses OS cells’ proliferation by acting on proteins involved in the cell cycle progression [29,30]. Particularly, Nagao-Kitamoto et al. showed that RNAi-mediated knockdown of GLI2 significantly reduces the migration, invasion, and lung metastasis of OS cells. These results indicate that GLI2 plays a role not only in the proliferation of osteosarcoma cells but also in their invasion and metastatic potential [27]. Regarding GLI1, Nakamura and collaborators demonstrated that arsenic trioxide (ATO) induces a decrease in Hh target gene expression levels (PTCH1, GLI1, and GLI2) in human OS cell lines; in particular, they revealed that ATO inhibits GLI transcription, thus preventing OS progression and growth in vitro [31].

Curcumin (CUR), a natural polyphenol derived from the turmeric plant (Curcuma longa), has attracted considerable attention for its anti-cancer properties [32]. In various cancer models—breast cancer cells [33], gastric cancer cells [34], medulloblastoma [35]—CUR has been shown to inhibit the Hh pathway by downregulating the expression of SHh, PTCH1, and GLI1/GLI2. These effects lead to reduced cell proliferation, invasion, and migration [33,34,35]. For example, in breast cancer, CUR inhibits the Hh pathway, suppressing the epithelial–mesenchymal transition (EMT), reducing its metastatic potential [33]. Similarly, in gastric cancer, CUR decreases the expression of Hh and Wnt pathway components, demonstrating its ability to modulate multiple signaling pathways to exert anti-tumor effects [36].

Given the critical role of the Hh pathway in OS and the promising effects of CUR in modulating this pathway in other cancers [19,20,21,25,26,32,33,34,35], we propose, for the first time, the use of CUR as a therapeutic agent to target the Hh pathway in OS. This novel approach aims to counteract OS progression, reduce metastasis, and overcome chemoresistance. By inhibiting the Hh pathway, CUR could potentially serve as an adjunct therapy, improving the outcomes for OS patients and providing a new avenue for clinical treatment strategies. This manuscript explores the mechanistic insights and therapeutic potential of CUR in modulating the Hh pathway in OS, offering a promising new direction in cancer therapy.

## 2. Results

### 2.1. Effect of Curcumin and Methotrexate on MG-63 Viability and Cytotoxicity

We conducted an MTT assay to assess the effects of Curcumin (CUR) and methotrexate (MTX) on the viability and cytotoxicity of the OS cell line (Table 1 A,B; Table S1). The results indicated that MTX [0.1 µM], [0.5 µM], [1 µM], and [1.5 µM] (Table 1A), and that CUR [0.1 µM] and [0.5 µM] (Table 1B) did not significantly impact the viability of MG-63 compared to the untreated group (NT). We observed that MTX [2 µM], [2.5 µM], and [3 µM], and CUR [1 µM] induced a reduction in cell viability, but not in a strong manner. We also revealed a strong reduction in cell viability with CUR [3 µM], [5 µM], [10 µM], and [20 µM] treatments, letting us consider these concentrations cytotoxic (Table 1B).

### 2.2. Effects of Curcumin and Methotrexate on Apoptosis in OS

**Figure 1:**
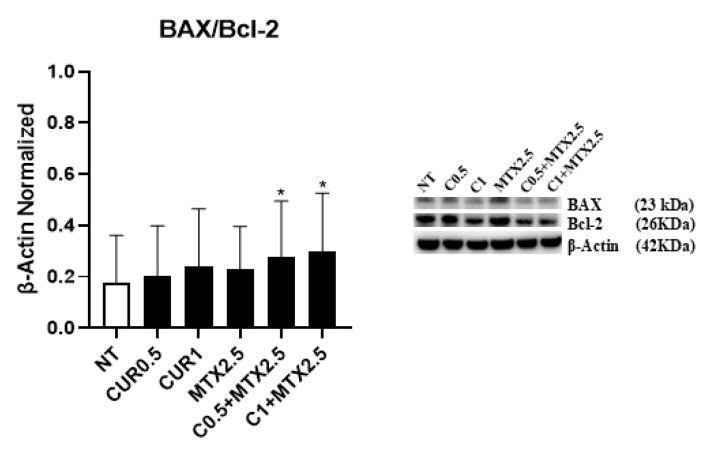
Effects of Curcumin and Methotrexate on apoptosis. BAX/Bcl-2 ratio protein expression levels in MG-63, determined by Western blot, starting from 15 μg of total lysate after 48 h of exposure to Curcumin (CUR) [0.5 µM and 1 µM] and Methotrexate (MTX) [2.5 µM], alone and in combination. The protein bands were detected using Image Lab Ink 6.1 software “BIORAD”, and the intensity ratios of immunoblots were quantified after normalizing with the housekeeping protein β-Actin. The graph represents the ratio between BAX and Bcl-2 as the mean ± SD. Student’s *t*-test was used for statistical analysis. * *p* ≤ 0.05 compared to non-treated (NT) MG-63 cells.

We explored the impact of CUR at concentrations of [0.5 μM] and [1 μM] and MTX at [2.5 μM], both individually and together, on apoptosis in MG-63 cells. Using Western blot analysis, we determined the ratio of expression levels between two key apoptotic proteins: BAX, a pro-apoptotic protein, and Bcl-2, an anti-apoptotic one. All treatments increased this ratio; notably, the combination treatments led to a statistically significant rise in the BAX/Bcl-2 ratio (Figure 1), thereby enhancing apoptotic effects. These results were further validated through an apoptosis cytofluorimetric assay, which showed an increased percentage of total apoptotic cells following combined treatments, which is statistically significant when MTX is combined with CUR [0.5 μM] (Table 2). Overall, these results demonstrate that the combined application of CUR at [0.5 μM] and [1 μM], together with MTX at [2.5 μM], promotes pro-apoptotic effects in the OS cell line.

### 2.3. Effects of Curcumin and Methotrexate on Hedgehog Signaling Pathway in OS

**Figure 2:**
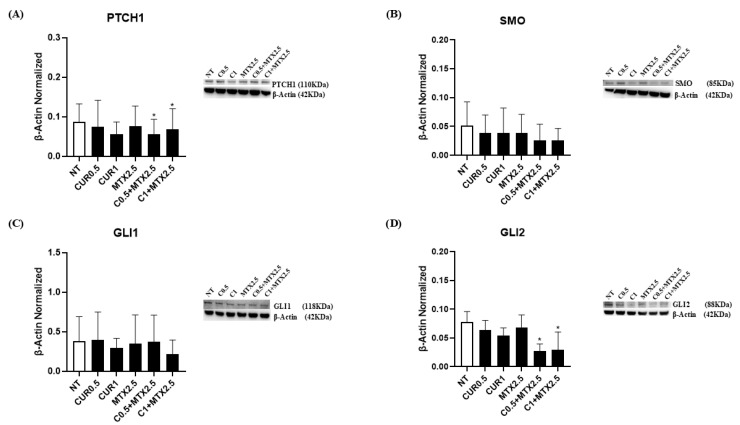
Effects of Curcumin and Methotrexate on Hedgehog signaling pathway. PTCH1 (**A**), SMO (**B**), GLI1 (**C**), GLI2 (**D**) protein expression levels in MG-63, determined by Western blot, starting from 15 μg of total lysates after 48 h of exposure to Curcumin (CUR) [0.5 µM and 1 µM] and Methotrexate (MTX) [2.5 µM] alone and in combination. The most representative images are displayed. The protein bands were detected using Image Lab Ink 6.1 software “BIORAD”, and the intensity ratios of immunoblots were quantified after normalizing with the housekeeping protein β-Actin, which is represented in the histograms as the mean ± SD. Student’s *t*-test was used for statistical analysis. * *p* ≤ 0.05 compared to non-treated (NT) MG-63 cells.

To investigate the effect of CUR [0.5 μM] and CUR [1 μM], alone or combined with MTX [2.5 μM], on the Hedgehog signaling pathway in OS, we assessed the protein expression levels of the receptors PTCH1 and SMO, as well as the transcription factor GLI2 in the MG-63 cell line by Western blotting. Both CUR concentrations [0.5 μM] and [1μM] led to a reduction in PTCH1, SMO, and GLI2 levels, although these reductions were not statistically significant. MTX [2.5 μM] alone showed a trend toward reducing these proteins, and when co-administered with CUR, both [0.5 μM] and [1 μM], both PTCH1 and GLI2 levels significantly decreased, while SMO only showed a reduction trend (Figure 2A,B,D). Regarding GLI1, we noticed a decreasing trend following the administration of CUR [0.5 μM] and [1 μM] when combined with MTX [2.5 μM] (Figure 2C). These findings suggest that CUR may exert anti-tumor effects in OS by modulating the Hedgehog signaling pathway.

### 2.4. Effects of Curcumin and Methotrexate on OS Cell Survival and Proliferation

**Figure 3:**
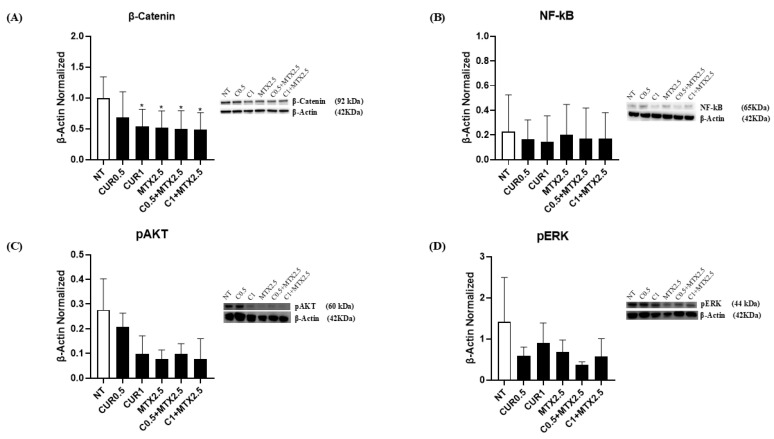
Effects of Curcumin and Methotrexate on OS cell survival and proliferation. β-Catenin (**A**), NF-kB (**B**), pAKT (**C**), and pERK (**D**) protein expression levels in MG-63, determined by Western blot, starting from 15 μg of total lysates after 48 h of exposure to Curcumin (CUR) [0.5 µM and 1 µM] and Methotrexate (MTX) [2.5 µM], alone and in combination. The most representative images are displayed. The protein bands were detected using Image Lab Ink 6.1 software “BIORAD”, and the intensity ratios of immunoblots were quantified after normalizing with the housekeeping protein β-Actin, which is represented in the histograms as the mean ± SD. Student’s *t*-test was used for statistical analysis. * *p* ≤ 0.05 compared to non-treated (NT) MG-63 cells.

By performing Western blotting, we examined the effects of CUR and MTX treatments on β-Catenin protein levels in the MG-63 OS cell line. β-Catenin is an oncogenic protein that plays a critical role in regulating cell–cell adhesion and is frequently overexpressed in tumors. Treatment with CUR at concentrations of [0.5 μM] and [1 μM], both alone and in combination with MTX [2.5 μM], led to a significant decrease in β-Catenin levels (Figure 3A). Additionally, we assessed the impact of these treatments on NF-kB protein levels in MG-63, a transcription factor implicated in uncontrolled proliferation and resistance to apoptosis in cancer cells. Both treatments, alone and combined, resulted in a reduction in NF-kB, although this reduction was not statistically significant (Figure 3B). Moreover, we analyzed the effects of both drugs on pAKT and pERK protein expression levels—two proteins involved in cell proliferation and whose expression is very increased in several types of cancers [37,38]. We did not observe any statistically significant variation after CUR [0.5 µM] treatment on pAKT protein expression; conversely, we revealed a strong reduction in the pAKT protein expression level after CUR [1 µM] and MTX [2.5 µM] administration. Moreover, the combined treatment also showed a marked decrease in pAKT (Figure 3C). Finally, we also evaluated the effects of both drugs on pERK and we demonstrated that also in this case, CUR [1 µM] and MTX [2.5 µM] treatments induced a reduction in pERK protein expression levels, which was more marked after MTX administration. The combination of CUR and MTX led to a reduction in pERK expression levels (Figure 3D). These results let us suppose that CUR and MTX could potentially be co-administered to exert anti-cancer effects by inhibiting cancer cell survival and proliferation.

### 2.5. Effects of Curcumin and Methotrexate on OS Cell Invasion and Migration

**Figure 4:**
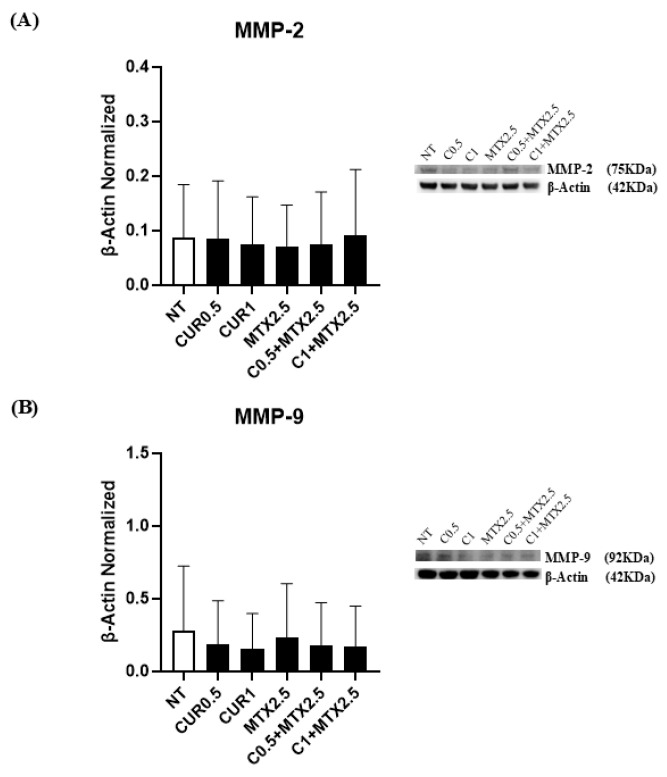
Effects of Curcumin and Methotrexate on OS cell invasion and migration. MMP-2 (**A**) and MMP-9 (**B**) protein expression levels in MG-63, determined by Western blot, starting from 15 μg of total lysates after 48 h of exposure to Curcumin (CUR) [0.5 µM and 1 µM] and Methotrexate (MTX) [2.5 µM], alone and in combination. The most representative images are displayed. The protein bands were detected using Image Lab Ink 6.1 software “BIORAD”, and the intensity ratios of immunoblots were quantified after normalizing with the housekeeping protein β-Actin, which is represented in the histograms as the mean ± SD. Student’s *t*-test was used for statistical analysis.

To assess the potential impact of CUR and MTX on cell invasion and migration, we examined the protein expression levels of matrix metalloproteinases MMP-2 and MMP-9. Treatment with CUR at concentrations of [0.5 μM] and [1 μM], particularly in combination with MTX [2.5 μM], showed a trend toward reduced expression of these proteins in MG-63 cells, which was more evident for MMP-9, although the results were not statistically significant (Figure 4A,B).

## 3. Discussion

The search for new therapeutic strategies against osteosarcoma (OS) has become one of the most important challenges today, given the severity, metastatic potential, and chemoresistance of the disease, and also the serious chronic adverse reactions resulting from the current therapeutic strategies actually in use [1,2,3,6,7,9,10,11]. Consequently, there is an urgent need to identify novel, effective therapeutic strategies with a better safety profile.

Several studies have been underway in recent years to discover other putative treatment strategies for OS, identifying other possible signaling pathways involved in OS pathogenesis. Intriguing studies include the investigation conducted by Punzo et al. on targeting the Cannabinoid Receptor 2 (CB2) and Transient Receptor Potential Vanilloid 1 (TRPV1) to induce anti-tumoral effects in OS [39,40]. In addition, proteasome inhibitors—like MLN2238 and Bortezomib—have shown efficacy in inducing apoptosis and reducing invasiveness in OS cells [40,41,42,43]. Interestingly, combining Bortezomib with CB2 and TRPV1 agonists enhances these actions and reduces side effects related to chemotherapy [40]. Immune-based therapies have also shown promising results, especially with the use of Mifamurtide, a drug approved in Europe for OS treatment involved in the activation of immune cells to control tumor progression [7,44]. It was demonstrated that Mifamurtide not only enhances immune-mediated anti-cancer effects but also exhibits anti-osteoporotic properties and reduces tumor vascularization [45,46,47]. Furthermore, antibody-based treatments targeting tumor cell surface proteins, such as Glembatumumab-vedotin, Trastuzumab, and Denosumab, have also been investigated in OS [48,49,50]. However, combining Denosumab with doxorubicin exacerbates chemotherapy’s effects, making it unsuitable for OS treatment [4,51,52]. Recent studies suggest iron chelators for OS therapy, even though their effectiveness varies; indeed, while Dp44mT shows promising anti-cancer activity, deferasirox (DFX) and eltrombopag (ELT) do not induce significant anti-tumoral effects on OS cell lines [53,54].

Therefore, given the importance of research into novel therapeutic strategies for OS treatment to reduce the debilitating side effects caused by chemotherapy, in fact, one promising area of research is targeting specific molecular pathways dysregulated in OS.

The investigation of the involvement of the Hedgehog (Hh) signaling pathway in cancer cell proliferation, survival, and metastasis is of particular scientific interest [13]. This pathway—crucial for embryogenesis, organ development, and tissue maintenance—includes several compounds, among them patched receptors (PTCH1 and PTCH2) [15], SMO [16,17], and GLI transcription factors (GLI1, GLI2, GLI3) [15]. Hh signaling pathway dysregulation is implicated in several kinds of cancers, including leukemia, basal cell carcinoma, and medulloblastoma [13,18]. Particularly, it is already reported that PTCH1, SMO, and GLI1/GLI2 are overexpressed in OS cell lines and patient samples, and that this increased expression is correlated with poor clinical outcomes [13,19,20,21,22,23,24,25]. Nagao et al. demonstrated the involvement of GLI2 in OS growth, proposing it as a putative novel therapeutic target for OS [24]. Effectively, they proved this hypothesis by observing the reduction in 143B and Saos-2 OS cell lines’ proliferation, thus inhibiting OS cell growth following *GLI2* knockdown [24]. Additionally, they also demonstrated that knockdown of GLI2 modulates cell cycle progression by reducing cyclin D1, pRb, and SKP2 protein expression levels, thus confirming once again the involvement of the Hh signaling pathway in the pathogenesis and progression of OS [24]. Interestingly, Lo et al. directed an intriguing molecular study about the involvement of the Hh signaling pathway in OS by observing an increased genic expression of the compounds of this pathway, such as the ligand IHH and its targets, PTCH1 and GLI1, in about 43 OS samples [23]. Moreover, they found a tight relationship between the genic expression of IHH and PTCH1 in large tumors, suggesting a ligand-dependent activation. Instead, regarding the other Hh signaling compounds, GLI1, PTCH1, and SMO, a positive correlation was observed between them in the cases of small tumors, thus indicating a ligand-independent activation. Therefore—considering their results—it is interesting to note that both ligand-dependent and ligand-independent mechanisms promote the stimulation of the Hh signaling pathway in OS and, unfortunately, that the ligand-dependent one induced by high IHH levels is responsible for tumor growth [23].

Hence, considering the involvement of this pathway in cell proliferation, differentiation, and survival, it could be considered as a novel therapeutic target to counteract cancer progression and—more specifically—OS progression.

Moreover, recent studies indicate that Curcumin (CUR) has significant anti-cancer properties in several kinds of tumors [32]. Particularly, various research has reported that CUR acts as a chemotherapeutic drug by regulating different signaling pathways involved in OS growth, progression, and metastasis, suggesting it for OS treatment in addition to the chemotherapeutics actually in use, thus reducing the side effects normally associated with this kind of therapy [55,56,57]. Indeed, CUR is able to modulate different pathways normally involved in OS progression, among them Wnt/β-Catenin, RANK/RANKL, MAPK/ERK, PI3k/AKT, Notch, and, interestingly, ferroptosis [55,57]. Yuan et al. proposed the Nrf2/GPX4 signaling pathway as a novel target for CUR in in vitro and in vivo OS models, able to mediate ferroptosis, thus counteracting tumor progression [57]. Other different studies have been conducted on signaling pathways influenced and regulated by CUR [58]. An anti-apoptotic effect induced by CUR is reported, which activates the apoptotic effector protein Caspase 3 and determinates an increased expression of the pro-apoptotic protein BAX and a reduction in the anti-apoptotic protein Bcl-2 expression levels [58]. Moreover, it has also been reported that CUR modulates cell cycle progression by blocking cells in G1/S and G2/M phases, inhibiting OS cell proliferation.

Interestingly, recent studies have shown that CUR inhibits the Hh pathway in several other cancers, such as breast cancer, gastric cancer, and medulloblastoma, by downregulating the expression of SHh, PTCH1, and GLI1/GLI2, leading to reduced cell proliferation, invasion, and migration [33,34,35].

Therefore, considering the critical role of the Hh pathway in OS and the promising effects of CUR in modulating this pathway in other types of cancer, we propose using CUR as a therapeutic agent to target the Hh pathway in OS.

In particular, our study explored the effects of CUR and MTX—the chemotherapeutic agent frequently used in OS—on OS progression by evaluating the effects of their administration on apoptosis, Hh signaling modulation, cell survival, proliferation, invasion, and migration.

Firstly, we demonstrated that CUR and MTX administration, alone and particularly in combination, increased the BAX/Bcl-2 ratio in a strong manner in the MG-63 cell line. It is known that BAX is an important pro-apoptotic protein, while Bcl-2 inhibits apoptosis [13]; therefore, their ratio is indicative of an apoptotic event. The observed increased BAX/Bcl-2 ratio let us suppose that these drugs promote the apoptosis of OS cells. We supported these data by performing an apoptosis assay and revealing a trend toward an increase in the total apoptotic cell percentage, particularly due to the effects induced by MTX. Indeed, considering the effect of CUR administration, we could not observe any significant variation in the total apoptotic cell percentage, while—as expected—MTX led to a trend toward an increase in these percentages. Our results show that the pro-apoptotic effect is enhanced when MTX is administered in combination with CUR, with a greater increase in the percentage of total apoptotic cells, which is statistically significant when MTX is combined with CUR 0.5 µM. Therefore, these results let us suppose that the combined administration of CUR and MTX could be more effective in inducing pro-apoptotic effects in OS, thus letting us possibly hypothesize a synergistic effect between CUR and MTX.

**Figure 5:**
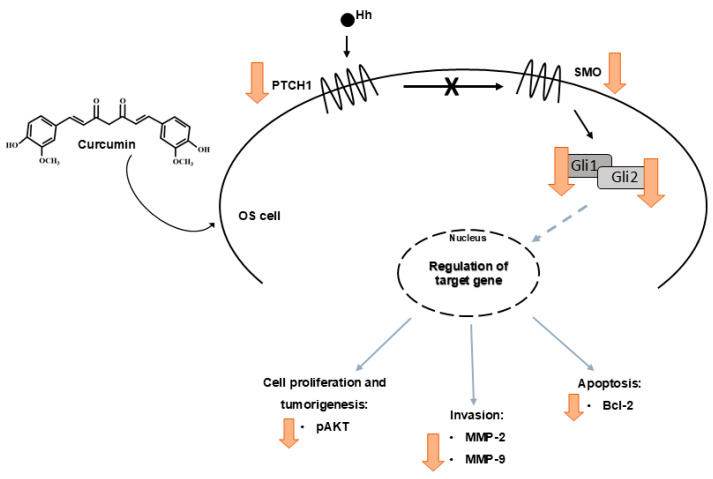
Diagrammatic presentation of the mechanism of action of Curcumin on Hedgehog signaling pathway in MG-63 cell line. CUR counteracts OS progression, inhibiting cell proliferation, tumorigenesis, and invasion and promoting apoptosis by modulating the component of Hh pathway.

Interestingly, we demonstrated for the first time in our knowledge that CUR is also able to modulate the protein expression levels of Hh signaling pathway components in OS, thus interfering with its progression (Figure 5). Particularly, we showed a reduction in the protein expression levels of both receptors PTCH1 and SMO, which was more evident when we co-administered CUR and MTX on MG-63, observing a statistically significant decrease in PTCH1. It is known that when the Hh signaling pathway is activated, the PTCH1 receptor is internalized and degraded, thus determining SMO activation [16,17]. Consequently, GLI transcription factors translocate into the nucleus and determine the transcription of genes responsible for cell proliferation, differentiation, and survival [15]. Therefore, the reduction in both PTCH1 and SMO protein expression levels we observed suggest that CUR is able to modulate the Hh signaling pathway by also improving the effect of MTX alone. We also confirmed this modulating property of CUR on the Hh signaling pathway by observing a strong reduction in the GLI2 protein expression levels, particularly when CUR was administered together with MTX. Conversely, these drugs did not affect in a statistically significant manner GLI1 expression levels, even though they are able to promote a stimulus to reduce the expression of this transcription factor. Since the activation of the Hh pathway is known to be involved in cancer progression, promoting the cell cycle progression, proliferation, and survival of OS cells, and also in chemoresistance [24], our results suggest that the CUR and MTX inhibiting effects on the Hh signaling pathway could be proposed as a promising novel therapeutic strategy able to inhibit cancer progression, reduce metastasis, and overcome chemoresistance.

Moreover, we further corroborated the anti-cancer effects of CUR and MTX in OS by observing the variation of β-Catenin protein expression levels. Effectively, it is known that the Wnt/β-Catenin system is involved in OS progression and, recently, it has also been proposed as a novel therapeutic target for OS management [59]. Our study revealed a strong reduction in the oncogenic protein β-Catenin expression levels both when the drugs were administered alone and when in combination, thus contributing to reduced OS cell survival.

We also decided to investigate the effects of CUR and MTX on NF-kB, considering the involvement of NF-κB signaling activation in promoting cell proliferation and cancer growth in several types of tumors [60]. However, we only observed a trend toward reduction, even though not in a statistically significant manner, specifically when CUR was administered alone. In order to strengthen the data concerning the effects of both drugs on proliferation, we also evaluated the protein expression levels of the phosphorylated and, thus, active forms of AKT and ERK (pAKT and pERK). Indeed, it is known that pAKT and pERK are involved in cell proliferation, and their levels are strongly increased in different tumor types [37,38]. We observed a strong reduction in pAKT when MTX was administered alone, and a less pronounced reduction when both drugs were combined. Regarding pERK, we only observed a trend to reduction when MTX was administered alone and in combination with both concentrations of CUR. Therefore, we can only hypothesize that CUR and MTX could contain OS cell line proliferation.

Finally, we investigated the CUR and MTX effects on OS cell invasion by investigating their effects on the expression levels of both metalloproteinases, MMP-2 and MMP-9. We demonstrated that both treatments were able to stimulate a slight reduction in MMP-2 protein expression levels, and a more pronounced decrease in MMP-9 expression levels, particularly when CUR was administered alone and in combination, although not in a statistically significant manner. These effects could be a consequence of the direct action of both drugs on MMP-2 and MMP-9 proteins or also an indirect effect mediated by the action of both drugs on the Hh signaling pathway, since it is known that it is involved also in the modulation of invasion mechanisms by regulating the expression of MMP-2 and MMP-9 [61].

In summary, our findings suggest that CUR shows potential as an effective therapeutic agent in OS by modulating the Hh signaling pathway and other critical cancer-related pathways. These actions are more effective when CUR is combined with MTX, letting us suppose a promising synergism between these two drugs. Even though other in vitro and in vivo studies are obviously needed to better understand and validate these already promising results, our findings open new avenues for research and clinical strategies to improve OS treatment outcomes and reduce the adverse effects associated with current therapies.

## 4. Materials and Methods

### 4.1. Cell Lines

Human OS cell line MG63 was purchased from ATCC and cultured in EMEM medium (Gibco Limited, Uxbridge, UK), supplemented with 10% fetal bovine serum (FBS) (Euroclone, Pero, Italy), 1% Non-Essential Amino Acids (NEAA) (Gibco Limited, Uxbridge, UK), 100 U/mL penicillin (Gibco Limited, Uxbridge, UK), 100 U/mL streptomycin (Gibco Limited, Uxbridge, UK), and 2 mM L-glutamine (Euroclone, Pero, Italy). Cells were cultured at 37 °C in a humidified atmosphere with 5% CO_2_. After 48 h, adherent cells were harvested using trypsin, washed, and counted on a microscope using a Bürker Haemocytometer, and 4.0 × 10^5^ cells per well were plated in a 6-well plate. Once 80% confluence was reached, Curcumin [0.5 μM] and [1 μM] and Methotrexate [2.5 μM] were added alone and in combination. Cells were collected after 48 h of incubation for protein extraction and Muse “Annexin V and Dead Cell Assay” (Millipore, Burlington, MA, USA).

### 4.2. Drugs and Treatments

Curcumin (CUR) was purchased from Sigma Aldrich (St. Louis, MO, USA) as powder, and was dissolved in pure ethanol and used in vitro at the following concentrations: 0.5 µM and 1 µM. Methotrexate (MTX) was purchased from Teva Italia (Milan, Italy) and was diluted in sterile water and used at a concentration of 2.5 µM. The concentrations of CUR and MTX were determined through concentration–response experiments and were those producing the strongest effect without altering cells’ viability (Table S2).

Non-treated cultured cells were maintained in incubation media during the relative treatment time with and without a vehicle (sterile water and pure ethanol). CUR was administrated every 24 h.

### 4.3. MTT Cell Viability and Cytotoxic Assay

We evaluated the viability of the MG-63 cell line after 48 h of treatment, using the colorimetric MTT assay and following the manufacturer’s guidelines (Elabscience, Houston, TX, USA). The analyzed concentrations were CUR [0.1 µM, 0.5 µM, 1 µM, 3 µM, 5 µM, 10 µM, 20 µM] and MTX [0.1 µM, 0.5 µM, 1 µM, 1.5 µM, 2 µM, 2.5 µM, 3 µM].

MTT, or 3-(4,5-dimethylthiazol-2-yl)-2,5-diphenyl tetrazolium bromide, can be reduced by certain mitochondrial dehydrogenases to form a purple crystalline product known as formazan. Formazan can be dissolved in DMSO, with its absorbance measured at approximately 570 nm. The rate of cell proliferation is indicated by the intensity of the color, with a darker color signifying faster proliferation and a lighter color indicating higher cytotoxicity. There is a direct linear correlation between the color intensity and the cell count.

### 4.4. Annexin V and Dead Cell Assay

After 48 h of treatment with CUR [0.5 μM] and [1 μM] and MTX [2.5 μM], both alone and in combination, we assessed the apoptosis of the MG-63 cell line using a fluorometric assay with the “Cell Dead and Annexin V Assay Kit” and the Muse cell analyzer. The Muse™ Annexin V and Dead Cell Assay employs Annexin V to detect phosphatidylserine (PS) on the membrane of apoptotic cells and uses 7-amino-actinomycin D (7-AAD) as a dead cell marker to indicate cell membrane integrity. In brief, 100 μL of a cell suspension (1 × 10^5^ cells/mL) was mixed with 100 μL of Muse™ Annexin V and Dead Cell Reagent and incubated for 20 min at room temperature in the dark. The results were automatically displayed and analyzed using “Muse 1.4 Analysis” software for data acquisition and analysis.

### 4.5. Western Blotting

Proteins were extracted from a treated and untreated MG-63 cell line using radio-immunoprecipitation assay (RIPA) lysis buffer (Millipore, Burlington, MA, USA) according to the manufacturer’s guidelines. BAX, Bcl-2, PTCH1, SMO, GL1, GLI2, β-Catenin, NF-kB, MMP-2, and MMP-9 proteins were identified in the total lysates from cell cultures via Western blotting. Membranes were incubated overnight at 4 °C with the following antibodies: anti-BAX antibody (1:100, Mouse, Santa Cruz Biotechnology, Dallas, TX, USA) (molecular weight: 23 KDa), anti-Bcl-2 (1:200, Mouse, Santa Cruz Biotechnology, Dallas, TX, USA) (molecular weight: 26 KDa), anti-PTCH1 (1:500, Rabbit. Elabscience, TX, USA) (molecular weight: 110 KDa), anti-SMO antibody (1:100, Mouse, Santa Cruz Biotechnology, Dallas, TX, USA) (molecular weight: 85 KDa), anti-GLI1 antibody (1:500, Rabbit. Elabscience, TX, USA) (molecular weight: 118 KDa), anti-GLI2 antibody (1:500, Rabbit, Proteintech, Rosemont, IL, USA) (molecular weight: 88 KDa), anti-β-Catenin (1:500, Mouse, BD Transduction Laboratories, San Jose, CA, USA) (molecular weight: 92 KDa), anti-NFkB (1:500, Mouse, Elabscience, TX, USA) (molecular weight: 65 KDa), anti-pAKT (1:1000, Mouse, Elabscience, TX, USA) (molecular weight: 60 KDa), anti-p-ERK (1:500, Rabbit, Elabscience, TX, USA) (molecular weight: 44 KDa), anti-MMP-2 antibody (1:1000, Rabbit, Elabscience, TX, USA) (molecular weight: 75 KDa), and anti-MMP-9 (1:500, Mouse, Invitrogen by Thermo Fisher, Waltham, MA, USA) (molecular weight: 92 KDa). Reactive bands were detected using chemiluminescence (Clarity Max Western ECL Substrate, BioRad, Hercules, CA, USA) on a CHEMIDOC Bio-Rad system (BioRad, Hercules, CA, USA). A mouse monoclonal anti-β-Actin antibody (1:500, Santa Cruz Biotechnology, TX, USA) (molecular weight: 42 KDa) was used to ensure comparable protein loading and served as a housekeeping protein. Images were captured, stored, and analyzed using “Image Lab Ink 6.1” software.

### 4.6. Statistical Analysis

Statistical analyses for all experiments were conducted using the Student’s *t*-test to assess differences between quantitative variables. We employed GraphPad Prism version 8.4.2 for this purpose. The experiments were run in triplicate (only for the apoptosis we performed six replicate), and the data are presented as mean ± SD. A *p*-value ≤ 0.05 was considered statistically significant.

## 5. Conclusions

In conclusion, our study highlights the potential of CUR as a therapeutic agent in the treatment of OS, particularly when combined with MTX. By targeting the Hh signaling pathway, CUR and MTX demonstrated significant pro-apoptotic effects, as evidenced by the increased BAX/Bcl-2 ratio and overall apoptosis in OS cells. Additionally, these agents reduced the expression of key Hh pathway components, such as PTCH1, SMO, GLI1, and GLI2. The combined administration of CUR and MTX also led to a notable decrease in the oncogenic protein β-Catenin and showed a promising trend toward reducing NF-kB and matrix metalloproteinases (MMP-2 and MMP-9), thereby inhibiting OS cell proliferation, survival, and invasion. Our findings suggest that CUR, especially in combination with MTX, could be a promising addition to OS treatment regimens, potentially improving patient outcomes and reducing the adverse effects associated with current therapies.

## Figures and Tables

**Table 1:** Effects of Curcumin and Methotrexate on viability and cytotoxicity of OS cell line. (**A**) Cell survival rate in MG-63 cells after 48 h of treatment with Methotrexate (MTX) at different concentrations (0.1 μM–0.5 μM–1 μM–1.5 μM–2 μM–3 μM). The results are presented as the mean percentage ± standard deviation percentage (SD). Student’s *t*-test was used for statistical analysis. * *p* ≤ 0.05 compared to non-treated (NT) MG-63 cells. (**B**) Cell survival rate in MG-63 cells after 48 h of treatment with Curcumin (CUR) at different concentrations (0.1 μM–0.5 μM–1 μM–3 μM–5 μM–10 μM–20 μM). The results are presented as the mean percentage ± standard deviation percentage (SD). Student’s *t*-test was used for statistical analysis. * *p* ≤ 0.05 compared to non-treated (NT) MG-63 cells.

(**A**) MTX Cell Survival Rate (%)	
NT	100
MTX [0.1 μM]	99.9 ± 10.7
MTX [0.5 μM]	99.8 ± 13.7
MTX [1 μM]	99.2 ± 13.0
MTX [1.5 μM]	86.5 ± 11.9
MTX [2 μM]	86.2 ± 3.8 *
MTX [2.5 μM]	84.1 ± 4.1 *
MTX [3 μM]	62.2 ± 3.2 *
(**B**) CUR Cell Survival Rate (%)	
NT	100
CUR [0.1 μM]	96.0 ± 3.5
CUR [0.5 μM]	96.4 ± 9.1
CUR [1 μM]	59.5 ± 11.3 *
CUR [3 μM]	2.6 ± 0.63 *
CUR [5 μM]	2.3 ± 0.99 *
CUR [10 μM]	1.5 ± 0.55 *
CUR [20 μM]	2.0 ± 0.42 *

**Table 2:** Effects of Curcumin and Methotrexate on apoptosis. Percentage of total apoptotic MG-63 cells revealed by Muse Annexin V and Dead Cell Assay after 48 h treatments with Curcumin (CUR) [0.5 µM and 1 µM] and Methotrexate (MTX) [2.5 µM], alone and in combination. The results are presented as the mean percentage ± SD of three independent experiments and were analyzed by Student’s *t*-test. * *p* ≤ 0.05 compared to non-treated (NT) MG-63 cells.

Percentage of Total Apoptotic Cells (%)	
NT	22.6 ± 2
CUR [0.5 μM]	23.1 ± 4
CUR [1 μM]	19.1 ± 2
MTX [2.5 μM]	29.9 ± 4
CUR [0.5 μM] + MTX [2.5 μM]	37.6 ± 6 *
CUR [1 μM] + MTX [2.5 μM]	38.4 ± 8

## Data Availability

The data presented in this study are available in this article (and supplementary material).

## Supplementary Materials

The following supporting information can be downloaded at https://www.mdpi.com/article/10.3390/ijms252011300/s1.
