# Supplementary material for: Curcumin and Methotrexate: A Promising Combination for Osteosarcoma Treatment via Hedgehog Pathway Inhibition
Source: Int J Mol Sci. 2024 Oct 21;25(20):11300. doi: 10.3390/ijms252011300 (PMC11509055; doi:10.3390/ijms252011300)
Supplement: Supplementary file 1 [file ijms-25-11300-s001.zip › Supplementary Table 1.pdf]

Supplementary Table 1. Effects of Curcumin and Methotrexate combined treatment on viability and cytotoxicity of OS cell line. Cell survival rate in MG-63 cells after 48h of treatment with Curcumin (CUR) at concentrations of 0.5μM and 1μM in combination with Methotrexate (MTX) at concentrations of 2.5μM. The results are presented as the mean percentage ± standard deviation percentage (SD). Student's t-test was used for statistical analysis. *p ≤ 0.05 compared to non-treated (NT) MG-63 cells.

**CUR and MTX Combined Treatment Cell Survival Rate (%)**	
NT	100
CUR [0.5μM] + MTX [2.5μM]	50.5 ± 3,1*
CUR [1μM] + MTX [2.5μM]	56,8 ± 1,8*
