# Supplementary material for: Curcumin and Methotrexate: A Promising Combination for Osteosarcoma Treatment via Hedgehog Pathway Inhibition
Source: Int J Mol Sci. 2024 Oct 21;25(20):11300. doi: 10.3390/ijms252011300 (PMC11509055; doi:10.3390/ijms252011300)
Supplement: Supplementary file 1 [file ijms-25-11300-s001.zip › Supplementary Table 2.pdf]

Supplementary Table 2. Dosing regimen table of Curcumin and Methotrexate on MG-63 cell line. *Group 1*: MG-63 NT; *Group 2:* MG-63 CUR 0.5 µM; *Group 3:* MG-63 CUR 1 µM; *Group 4:* MG-63 MTX 2.5 µM; *Group 5:* MG-63 CUR 0.5 µM + MTX 2.5 µM; *Group 6:* MG-63 CUR 1 µM + MTX 2.5 µM. Group 1 (NT) is the negative controls, where no treatment was administered. Curcumin-only groups (low and high doses) received Curcumin at two concentrations (0.5 µM and 1 µM) every 24 hours. Methotrexate group received a single dose of Methotrexate (2.5 µM). Combination treatment groups received Curcumin (either 0.5 µM or 1 µM) every 24 hours, along with a single dose of Methotrexate. N/A: not applicable.

Dosing Regimen

Experimental Group	Treatment	Curcumin (CUR) Dose	Methotrexate (MTX) Dose	Treatment Frequency	Duration	Changes in regimen
**Group 1 (NT)**	No Treatment	N/A	N/A	N/A	48 hours	No treatment
**Group 2 (CUR 0.5)**	Curcumin (Low Dose)	0.5 µM	N/A	Every 24 hours	48 hours	N/A
**Group 3 (CUR 1)**	Curcumin (High Dose)	1 µM	N/A	Every 24 hours	48 hours	N/A
**Group 4 (MTX 2.5)**	Methotrexate	N/A	2.5 µM	Single dose	48 hours	N/A
**Group 5 (CUR 0.5 + MTX 2.5)**	Curcumin + Methotrexate	0.5 µM	2.5 µM	CUR every 24 hours, MTX single dose	48 hours	No change from initial doses
**Group 6 (CUR 1 + MTX 2.5)**	Curcumin + Methotrexate	1 µM	2.5 µM	CUR every 24 hours, MTX single dose	48 hours	No change from initial doses
